# Inflammatory Bowel Disease Infusion Therapy Adherence in a Rural Pediatric Population

**DOI:** 10.7759/cureus.36753

**Published:** 2023-03-27

**Authors:** Laura R Hernandez Garcia, Zainab Shams, Alexa Magner, Katherine Webster, Stephanie Thompson, Pratikkumar P Patel

**Affiliations:** 1 Medical School, West Virginia University School of Medicine, Charleston, USA; 2 Medical School, West Virginia School of Osteopathic Medicine, Charleston, USA; 3 Institute of Academic Medicine, Charleston Area Medical Center, Charleston, USA; 4 Pediatric Gastroenterology, West Virginia University, Charleston, USA

**Keywords:** ulcerative colitis, crohn’s disease, pediatric ibd, barriers to infusions, infusion therapies, ibd therapy

## Abstract

Introduction: Biologic therapy is often used in patients with inflammatory bowel disease (IBD), which includes Crohn’s Disease (CD) and ulcerative colitis (UC). While biologic therapy improves outcomes, it is dependent on strict compliance for optimal benefit. Limited information is available to describe IBD infusion therapy compliance and adherence barriers in a rural, geographically dispersed pediatric population.

Methods: Parents/guardians and patients (aged 0-21 years) with a diagnosis of IBD and scheduled biologic therapy infusions were offered a survey consisting of a mix of multiple-choice and open-ended questions. Surveys were offered via in-person paper format or telephone.

Results: Of the 27 pediatric patients completing the survey, the mean age was 14 years old (SD 3.7 years) with 19 patients having CD and eight patients with UC. The results showed that more than half of the patients (59%) had to reschedule, miss, or delayed their infusion therapy at least once. Therapy compliance was maintained as patients were able to reschedule a new appointment within two weeks. The most common reasons for missing appointments were forgetfulness and school conflicts. Patients wanting to maintain health and avoid flare-ups were reported as key drivers for therapy.

Conclusion: Pediatric patients in rural and geographically disperse areas continue to have long commutes and other barriers to IBD specialty care. Forgetfulness and school activities were reported as barriers to biological therapy adherence. Protective factors including knowledge of therapy health benefits, parental involvement, and staff support can help maintain high adherence rates in this population.

## Introduction

Inflammatory bowel disease (IBD) includes Crohn’s disease (CD) and ulcerative colitis (UC). Treatment approaches vary depending on patient age and disease severity. Biologic therapy (such as infliximab, adalimumab, vedolizumab, ustekinumab, and other biosimilars) has been shown to significantly increase remission rates, improving the medical, physical, and mental well-being of children [1]. Depending on the type of biologic agents, some require IV infusions, which are typically given at infusion center facilities or at home by skilled staff.

Biologic therapy requires meticulous follow-up, as patients who are non-adherent experience increased relapses and hospitalizations leading to greater health costs [2]. Compliance with IBD therapies is often suboptimal. A study demonstrated initial infliximab infusion adherence to be 78% in adult patients, but maintenance adherence was significantly lower at 31% [3]. In contrast, a study with pediatric patients with IBD found greater than 90% adherence to infliximab infusions with the high therapy adherence hypothesized due to family and clinical staff support [1]. However, further research is needed to determine the specific factors contributing to treatment adherence. Factors associated with nonadherence to oral maintenance therapy in adolescents have been reported to be forgetfulness, location, scheduling conflict, family dynamics, psychosocial issues, lack of confidence in the treatment plan, and symptom reduction [2].

Our study focused on the pediatric population served by a regional, tertiary health care system with a dispersed patient population background, ranging from urban to rural regions in central Appalachia. The hospital has a large catchment area of approximately 12,000 square miles and serves the southern half of the state of West Virginia. This study determined adherence rate and barriers to IBD infusion therapy, with the goal to optimize adherence. Additionally, our goal was to study the pediatric rural population to identify unique barriers present in this population and to provide a groundwork for future initiatives to improve the health of pediatric patients with IBD living in geographically dispersed regions.

## Materials and methods

The study design was observational and descriptive to determine pediatric IBD infusion therapy adherence and barriers via the surveyed patients or caregivers. The study was conducted at the Infusion Center/Children’s Cancer Center (CCC) located at Charleston Area Medical Center Women and Children’s Hospital, Charleston, West Virginia, United States. All aspects of the study were approved by the Charleston Area Medical Center/West Virginia University-Charleston Institutional Review Board (approval number: 21-754).

All patients aged 0-21 years old with a diagnosis of IBD scheduled to receive maintenance biologic therapy intravenous infusions at the CCC center between February 1, 2022, and October 31, 2022, were invited to participate in this study. Patients who were no longer scheduled to receive therapy at the infusion center or were older than 21 years of age at the time of the survey were excluded from this study. 

The study utilized a non-validated survey designed by study investigators, which consisted of 16 questions regarding treatment adherence and barriers to compliance. In addition, the survey contained questions regarding patient demographics including age, sex, IBD type, and insurance status. The typical survey completion time was less than 10 minutes. If the patient was 18 years or older, then he/she gave consent and completed the survey. If the patient was between the ages of 13 and 17 years, then a parent/guardian gave consent and either the parent/guardian or adolescent patient completed the survey. If the patient was younger than 13 years, then a parent/guardian provided consent and completed the survey. 

The survey was offered via in-person paper format or by telephone to patients and/or parents/guardians. During a span of nine months, a cover letter explaining the study and a paper version of the survey were provided to the patient and parent/guardian upon arrival at CCC at a regularly scheduled infusion appointment. For patients/parents that did not complete the survey questionnaire in person, up to three attempts were made to contact participants via telephone. Phone numbers were obtained from CCC records. Documented verbal consent was obtained over the phone. 

The study’s primary outcome of treatment adherence was defined as maintaining regularly schedule appointments with missed infusions being received within two weeks of the originally scheduled date. A secondary outcome of interest included participant-reported barriers to maintaining IBD infusion therapy appointments. IBM SPSS Statistics for Windows, Version 19.0 (Released 2010; IBM Corp., Armonk, New York, United States) was used to analyze data. Basic descriptive statistics, such as means and standard deviations for continuous variables and proportions and frequencies for categorical variables, were used to analyze survey answers. Textual responses to open-ended questions were coded for common themes with frequencies reported. 

## Results

We invited the 38 pediatric patients, or their parents/guardians, scheduled to receive biological infusions for the IBD treatment during the nine-month study period to complete the survey. A total of three patients refused participation, six patients had no contact, one patient was older than 21 years of age, and one patient had incomplete documentation of consent; 27 patients completed the survey. 

The mean patient age was 14 (SD 3.7) years with a range of 8-20 years. Sex distribution was near equal, with 52% of patients male and 48% female. Within the IBD diagnosis, 19 patients had CD and eight had UC. Insurance coverage included public (Medicaid, Children's Health Insurance Program (CHIP)) in 12 patients and private/commercial in 11 patients; one patient was self-pay and three patients did not report insurance type. Additionally, 20 patients (70%) reported living more than 30 minutes from the infusion center, with two of those patients driving more than two hours to reach this specialty care. Fifty-two percent of the surveys were completed by the parent/guardian and 44% by the infusion recipient. 

The results showed that more than half of the patients (59%) had to reschedule, miss, or delayed their infusion therapy at least once (Table 1). The patients received the same medication with no difference in the frequency of infusions. Electronic health record (EHR) review of participants showed that 100% of patients with missed infusions rescheduled treatments within two weeks, maintaining therapy adherence. None of the participants required hospitalizations due to IBD exacerbation. 

**Table 1:** Survey Questions and Responses

Question	Responses	Frequency (n=27)
1. Have you ever missed, rescheduled, or delayed any scheduled infusions?	No	11 (41%)
	Yes	16 (59%)
If yes, how many infusions?	1-2	13 (48%)
	3-4	2 (7%)
	>5	0
	Missing	1 (4%)
2. Has the cost of infusion therapy ever caused you to miss or delay an infusion?	No	26 (96%)
	Yes	1 (4%)
3. Do you believe infusion therapy is beneficial to your health?	No	0
	Yes	26 (96%)
	Missing	1 (4%)
4. Have you ever missed, rescheduled, or delayed an infusion due to forgetfulness?	No	23 (85%)
	Yes	4 (15%)
5. Do you or your family set reminders?	No	6 (22%)
	Yes	21 (78%)
If yes, where/how?	Paper/phone calendar	20
6. Do you have any other chronic health conditions?	No	23 (85%)
	Yes	4 (15%)
If yes, do they influence your attendance for infusions?	No	2
	Yes	1
	Missing	1
7. Any infections in the past 12 months?	No	24 (89%)
	Yes	2 (7%)
	Missing	1 (4%)
If yes, did they cause you to miss/delay/reschedule an infusion?	No	2
	Yes	0
8. How far is your residence from the Children’s Cancer Center?	<30 mins	7 (26%)
	31-60 mins	11 (41%)
	61-120 mins	7 (26%)
	>120 mins	2 (7%)
9. Has the distance caused you to miss/reschedule/delay infusions?	No	26 (96%)
	Yes	1 (4%)
10. Are you (the patient) in school?	No	2 (7%)
	Yes	25 (93%)
If yes, have your school commitments interfered with infusion attendance?	No	21
	Yes	4
11. Do you (the patient) work?	No	22 (81%)
	Yes	5 (19%)
If yes, have your job commitments interfered with infusion attendance?	No	4
	Yes	1
12. Who accompanies infusion recipient to appointments (Choose all that apply)?	No one	2 (7%)
	Mother	22 (81%)
	Father	17 (63%)
	Grandparent	5 (19%)
	Sibling	3 (11%)
	Other	1 (4%)
Has their availability ever impacted your infusion attendance?	No	25 (93%)
	Yes	2 (7%)
Has their employment/school ever impacted your infusion attendance?	No	25 (93%)
	Yes	2 (7%)
Has their health ever impacted your infusion attendance?	No	26 (96%)
	Yes	1 (4%)

The survey contained the open-ended question “What has prevented or prevents you from making it to your infusions as scheduled?” From reported responses, infusion cost, other chronic conditions, and work responsibilities did not appear to be drivers of missed and reschedule appointments. The most common reasons for missed appointments were forgetfulness and schedule issues due to school attendance, each occurring in four patients. 

The survey contained three additional open-ended questions. For the question “What motivates or encourages you to come to infusion appointments as scheduled?”, all 25 patients' responses were related to maintaining health, and included “To stay healthy”, “It helps me from getting ill”, “To keep my disease under control”, and similar. For the question “Does the presence of ancillary services such as dietician and child life, motivate or impact your infusion attendance?”, only six out of the 22 respondents replied in the affirmative. For the last question “What would help increase attendance for scheduled infusions?”, of the 17 respondents, nine replied “nothing”, four citied reminders prior to the appointment, one requested a closer location, one cited transportation reimbursement, and one asked for more convenient hours (i.e., in the evening, weekend). 

## Discussion

Our study examined the barriers to receiving IBD infusion treatment in a rural, geographically dispersed pediatric population. For patients with IBD receiving infusion therapy, compliance is crucial for optimal clinical outcomes. Patients with low adherence rates experience more acute episodes of the disease and hospitalizations [1]. While we found that more than half of patients missed or delayed an appointment, they were rescheduled and attended quickly, maintaining a 100% maintenance adherence rate. 

Patients living in rural communities have additional challenges in maintaining appointments and receiving IBD specialty care. They often must travel two to three times farther to receive medical care [4], with the majority of our study’s patients having to commute more than 30 minutes for appointments. Furthermore, patients living in rural communities face health disparities. It has been reported in both Canada and the United States that rates of IBD-related office visit rates were lower for rural patients as compared to urban cohorts, while rates of hospitalizations and emergency department visits were greater [5,6]. The majority of our patients reported not missing appointments due to a long commute. This response from patients emphasizes the willingness to drive long distances for effective IBD treatment. However, further research into optimal methods of providing pediatric gastroenterology care to rural patients should be explored and may include remote clinics and telehealth. 

The survey results showed additional barriers that could influence our patients to miss or delay appointments. One identified barrier in our study was missing infusion therapies due to conflicts with school commitments. Attendance at school is one known social factor affected in patients with IBD. Researchers have previously described that approximately 60% of students had prolonged absences from school due to their IBD, and in turn, approximately 80% of those students thought they underachieve in school testing due to their health [7]. Despite documented accommodations and excused absences, apprehension about missing school or school work can remain and might explain the ongoing barrier of school conflicts described in our patient sample. This could be addressed at the school level by increasing awareness of the staff. 

Patients reported forgetfulness as a common reason for missing appointments. Adolescents with IBD have been found to have lower adherence rates than young children. Because this population often has more autonomy and a desire to “fit in”, it has been reported that “forgetting “and “interfering with other activities” are the main reasons for medical non-adherence [2,8]. Comparable results are seen in maintaining routine clinic appointments in the adolescent population as patients report forgetfulness (35%) as the major reason for missing appointments [9]. The addition of call reminders from a general adolescent health clinic in a randomized control trial showed a decreased non-attendance rate from 20% to 8% [9]. At the time of the study, reminder phone calls were not current practice in our clinic but a planned, future change in care. 

Although we describe some of the challenges experienced by our patient population, the adherence rate was maintained at 100% during this time. Our study results are consistent with the high rates of adherence described in the pediatric populations, which are believed to be related to parental involvement and clinical staff support [1]. The current workflow in our center aims to support our patients. Our staff promptly communicates with families via the phone when the patient missed an appointment to reschedule. Patients additionally interact with the same staff providing a sense of continuity of care. Additionally, we found a high emphasis of the patients and/or caregivers on reporting and acknowledging the benefits of infusion therapy on maintaining health. Others have found that medication knowledge is associated with increased oral medication adherence in adolescents with IBD [10]. Thus, patient education on treatments and benefits may assist in maintaining optimal IBD therapy compliance. 

Our study has some potential limitations, including a small sample size with participants mostly living in rural areas. A larger sample size might be needed in further research to consider this data representative of the general pediatric IBD population. Additionally, selection bias may have occurred due patients not participating in the survey having different experiences with missed infusions. 

## Conclusions

This study focused on the major barriers experienced by pediatric patients in maintaining IBD infusion therapy appointments. Our study showed unique challenges experienced by rural pediatric population including long travel times, conflicts with school attendance, and forgetfulness. The addition of call reminders, increasing disease awareness at school level and targeted care delivery strategies for rural patients may be useful in maintaining treatment adherence. Our study also highlights the protective factors of knowledge of treatment’s health benefits, parental involvement, and staff support which have been associated with high rates of adherence in the pediatric population.
